# Clinicopathological findings, treatment response and predictors of long-term outcome in a cohort of lupus nephritis patients managed according to the Euro-lupus regime: a retrospective analysis in Sri Lanka

**DOI:** 10.1186/s13104-017-2402-6

**Published:** 2017-02-02

**Authors:** Nalaka Herath, Neelakanthi Ratnatunga, Kosala Weerakoon, Abdul Wazil, Nishantha Nanayakkara

**Affiliations:** 1 Nephrology Unit, Teaching Hospital, Karapitiya, Galle, Sri Lanka; 2 Department of Pathology, Faculty of Medicine, University of Peradeniya, Peradeniya, Sri Lanka; 3 Department of Parasitology, Faculty of Medicine and Allied Sciences, Rajarata University of Sri Lanka, Saliyapura, Sri Lanka; 4 Nephrology Unit, Teaching Hospital, Kandy, Sri Lanka

**Keywords:** SLE, Lupus nephritis, Sri Lanka, Remission, Relapses

## Abstract

**Background:**

Despite the improvement in survival of patients with lupus nephritis (LN) globally, there is sparse data from Sri Lanka (SL). The current study aims to describe the clinicopathological findings, treatment response and predictors of long-term outcome of patients with WHO class III–IV LN in SL, managed according to the Euro-lupus regime.

**Results:**

Of 72 patients, 64 were females. In half of them, LN was diagnosed within the 1st year of the illness. The most common presenting feature was sub-nephrotic proteinuria. Sixteen and twenty patients had nephrotic syndrome and abnormal renal function respectively at the time of diagnosis. Fifty-four patients (75%) responded to the Euro-lupus regimen [CR, 20 (28%); PR, 34(47%)]. Later at 6 months, 65 patients (90%) achieved remission [CR, 31(43%); PR, 34 (47%)]. Seven patients experienced treatment failure. During the total duration of follow up, 54 patients remained in complete or partial remission, 26 developed renal relapses, and 19 suffered severe infective episodes. Renal relapses were more common in people who achieved partial remission than complete remission. The long term renal outcome was not associated with age, sex, severity of proteinuria, class of LN or initial renal function. Patients who achieved remission at 6 months had a good long-term outcome.

**Conclusions:**

The demographic and clinical features of WHO class III and IV LN in Sri Lankan patients were similar to that reported in the global literature. 75% of patients responded to the Euro-lupus regimen. Therefore, this regime is a suitable initial regimen for LN patients in SL. Good long-term renal outcome can be predicted by early response to therapy. Further studies are necessary to explore better treatment options for patients who fail to achieve remission during initial therapy.

## Background

Systemic lupus erythematosus (SLE) is an autoimmune disease which may give rise to multiple organ involvement because of immune complex deposits. Lupus nephritis (LN) is a notable complication of SLE occurring in 33–55% of cases leading to high morbidity and mortality [1–3]. Asymptomatic proteinuria, nephritic syndrome, nephrotic syndrome and rapidly progressive glomerulonephritis are some important clinical manifestations of LN. In America and Europe, the ten year renal survival rate has improved to 80–90% with the implementation of current immunosuppressive regimens [4, 5]. Literature on LN in Sri Lanka (SL) is very limited. One observational study carried out in SL showed that 69% of patients developed renal involvements during a follow up of 3 years. The majority of them had focal or diffuse proliferative glomerulonephritis [6]. The current study aims to describe the clinicopathological findings, treatment response and predictors of long-term outcome of patients with WHO class III–IV LN in SL, managed according to the Euro-lupus regime.

## Methods

### Patient selection

This is a retrospective analysis of the demography, clinical features, response to treatment and other complications in 72 Sri Lankan patients with renal histology confirmed WHO class III and IV lupus nephritis (Class III; N = 18, Class IV; N = 54) followed up at the Teaching Hospital, Kandy during the period January 2005–December 2011. SLE was diagnosed according to the criteria given by American College of Rheumatology. Patients who had pre-existing chronic kidney disease, pregnancy, previous malignancy and diabetes mellitus were excluded from the study. The study was approved by the Ethical Review Committee of the University of Peradeniya and written informed consent was obtained from all patients.

### Treatment received

All patients (n = 72) had been initially managed according to the Euro-lupus regime with low dose IV Cyclophosphamide pulses (6 pulses of IV Cyclophosphamide at a fixed dose of 500 mg fortnightly) and tapering dose of Prednisolone [7]. All patients had received 3 daily pulses of 500 mg of IV Methyl Prednisolone. A dosage of 1 mg/kg/day of Prednisolone had been given for 4 weeks, to critically ill patients, patients with renal impairment, nephrotic syndrome or severe extra-renal disease. Thereafter, the dosages were tapered by 10 mg of Prednisolone every month. All the other patients had been given a dose of 0.5 mg/kg/day of Prednisolone for 4 weeks. The dosage was tapered by 5 mg of Prednisolone every month. In all patients, glucocorticoid therapy was maintained at 7.5–10 mg of Prednisolone per day. After achieving complete or partial remission, they had been treated with Azathioprine (1.5–2 mg/kg) (n = 54, 75%) and maintained on a low dose of Prednisolone. Treatment failure patients after initial Euro-lupus regime, were treated with Mycophenolate Mofetil (MMF 1 g/day) (n = 18, 25%) and low dose Prednisolone.

### Definitions

Treatment failure was defined as any of the following 3 features:

1. Absence of a primary response
2. For patients with a baseline serum creatinine level >1.3 but <2.6 mg/dl, absence of a primary response was defined as failure of the serum creatinine level to decrease to <1.3 mg/dl at 6 months.
3. For patients with a baseline serum creatinine level >2.6 mg/dl, absence of a primary response was defined as failure of the serum creatinine level to improve by 50% at 6 months.
4. For patients with nephrotic syndrome without renal impairment (serum creatinine level <1.3 mg/dl), absence of a primary response was defined as the persistence of nephrotic syndrome at 6 months.
5. A glucocorticoid-resistant flare (defined as a severe flare that did not respond to a 1-month increase in the glucocorticoid dosage)

A severe flare was defined as one of the following 3 features: 33% increase in serum creatinine level within a 1-month period, development of nephrotic syndrome, or severe systemic disease (severe systemic disease defined as central nervous system disease, thrombocytopenia, haemolytic anaemia, lupus pneumonitis, lupus myocarditis, extensive skin vasculitis, or serositis not responding to low-dose glucocorticoid). Benign renal flares were defined as appearance of sub-nephrotic proteinuria without increase in serum creatinine level or severe systemic disease.

3. A doubling of the serum creatinine level over the lowest value reached at any time during the follow up and confirmed on 2 consecutive visits 1 month apart.

Complete remission (CR) was defined as 24-h urinary protein level less than 0.3 g/day and normal renal function (serum creatinine level <1.3 mg/dl) with an inactive urinary sediment.

Partial remission (PR) was defined as a urinary sediment with less than 10 red blood cells per high-power field, ≤25% increase in baseline creatinine, and ≥50% reduction in baseline proteinuria to ≤1.5 g/day.

### End points

Renal functions had been assessed on a regular basis monthly in all patients up to 1 year. However, further follow up was possible in only 61 patients as 11 were lost to follow up. The total duration of follow up varied from 1 to 6 years. The primary end point was treatment failure, which was defined as one of the following 3 features: absence of a primary response after 6 months of therapy, occurrence of a glucocorticoid-resistant flare, or a doubling of the serum creatinine level. Secondary end points were the rate of renal remissions and the number of severe flares.

### Statistical analysis

Results are expressed as means and standard deviations (SD) when presenting summary measures of continuous variables. Categorical variables are presented as numbers and percentages. Data analysis was done using Minitab (version 14) statistical software by means of measures of central tendencies and appropriate application of parametric and non-parametric statistics. Determination of different categories and number of patients were done as per the definitions given above. Association of different parameters with renal outcomes (CR, PR) was achieved by using the Student’s *t* test (age), Chi square test (level of proteinuria, initial kidney function, etc.) and Fisher’s exact test (gender, number of relapses etc.). P values of less than 0.05 were regarded as statistically significant.

## Results

Of 72 patients, 64 (89%) were females and the mean age of the total cohort was 28 years (range 13–60 years). In 37(51%) patients, LN was diagnosed within one year following the diagnosis of SLE. The commonest presentation was sub-nephrotic proteinuria (n = 56, 78%). Sixteen (22%) presented with nephrotic syndrome and 20 (28%) had abnormal renal function (serum creatinine >1.3 mg/dl).

Fifty-four patients (75%) responded to the Euro-lupus regimen [CR, 20 (28%); PR, 34 (47%)]. Later at 6 months, 65 patients (90%) achieved remission [CR, 31(43%); PR, 34 (47%)]. Seven patients (10%) experienced treatment failure. Of them three patients (4%) progressed to end stage renal disease and four patients (6%) had experienced doubling of serum creatinine. Eleven patients (15%) were lost to follow up. There was no significant difference between CR and PR in relation to the class of lupus nephritis at 1 year or during total duration of follow up (Fisher’s exact test; P values = 0.401 and 0.301 respectively) (Table 1).

**Table 1:** Summary of the renal outcome at different time points

Description	Number of patients followed up at different time points (n (%))											
	Biopsy class III (n = 18)				Biopsy class IV (n = 54)				Total (n = 72)			
	3 months	6 months	1 year^a^	Current status^b^	3 months	6 months	1 year	Current status	3 months	6 months	1 year	Current status
CR	13 (72)	13 (72)	13 (72)	13 (72)	15 (28)	18 (33)	28 (52)	27 (50)	20 (28)	31 (43)	41 (57)	40 (56)
PR	5 (28)	5 (28)	5 (28)	2 (11)	21 (39)	29 (54)	19 (35)	12 (22)	34 (47)	34 (47)	24 (33)	14 (19)
Treatment failure	0	0	0	0	18 (33)	7 (13)	7 (13)	7 (13)	18 (25)	7(10)	7 (10)	7 (10)
Lost follow up	0	0	0	3 (17)	0	0	0	8 (15)	0	0	0	11 (15)
Total	18 (100)	18 (100)	18 (100)	18 (100)	54 (100)	54 (100)	54 (100)	54 (100)	72 (100)	72 (100)	72 (100)	72 (100)

*CR* complete remission, *PR* partial remission

^a^ Fisher’s exact test between CR and PR in relation to class of lupus nephritis at 1 year; P value = 0.401

^b^ Fishers exact test between CR and PR in relation to class of lupus nephritis at present; P value = 0.301

The total number of relapses during follow up was 26 (36%). A higher proportion of relapses were seen among the patients who achieved PR than CR (P < 0. 05). Long term renal outcome (CR, PR) as per the number of patients available at present (current status), was not associated with age, sex, level of proteinuria or kidney function at diagnosis (Table 2). Patients who achieved CR or PR with immunosuppressive therapy at 6 months showed good long-term outcome (P value = 0.003). Severe infections were reported in 19 patients in our study.

**Table 2:** Association of long term renal outcome with clinical parameters

Description	Number of patients followed up [n (%)]		P value
	CR (n = 53)	PR (n = 14)	
Mean age [years (SD)]	27.6 (9)	28.2 (10)	0.672^a^
*Gender*			
Male	6 (11)	0	0.330^b^
Female	47 (89)	14 (100)	
*No of relapses*			
One	15 (28)	1 (7)	0.004^b^
Two	1 (2)	4 (29)	
*Level of proteinuria at the time of diagnosis*			
Nephrotic	11 (21)	1 (7)	0.435^b^
Sub-nephrotic	41 (77)	13 (93)	
*eGFR at the time of diagnosis*			
Low	10 (19)	3 (21)	0.99^b^
Normal	43 (81)	11 (79)	

*CR* complete remission, *PR* partial remission

^a^ Unpaired t test

^b^ Fisher’s exact test

## Discussion

Renal involvement is often a cause of significant morbidity and mortality in SLE. In most studies, renal involvement was found in approximately one half of patients with SLE [1, 8]. The young age of onset of LN and female predominance in our series is comparable to other studies [4, 8, 9]. The majority had renal involvement within the 1st year of onset of SLE, as has been reported in other studies too [4, 8, 9].

There are some similarities of clinical and biochemical features in patients in our study and the Euro lupus nephritis trial (ELNT) [7]. In our study, 28% of patients had abnormal renal functions at diagnosis and 22% had nephrotic syndrome as the presenting feature and in the ELNT study, the figures were 22 and 28% respectively [7]. In both studies, patients had proliferative LN in one dominant race (our patient population was Sri Lankan and ELNT study mainly consisted of Caucasians). The rate of renal remission in the ELNT study was 71% which was similar to that observed in our patients (75%) after the Euro-lupus regimen. However; we achieved high renal remission of up to 90% which may be due to the introduction of different treatment regime for those who had treatment failure after initial treatments. Further studies are necessary to explore better treatment options for these patients with treatment failure.

The number of renal relapses in our study and ELNT were 36 and 27% respectively. More relapses were seen among the patients who achieved PR than CR. A majority of patients experienced renal flare, while they were on Azathioprine. Because a very selective and small number of patients were given MMF, we could not compare the effectiveness of MMF over Azathioprine. This needs further studies in SL.

Infections as a sequel to immunosuppressive therapy have been reported as a fairly common problem in our region [8, 10–12]. Severe infective episodes were reported only in 19 patients in our study. It is possible that the rate of infection is much higher than that mentioned here, due to under reporting of such episodes managed at local hospitals or by general practitioners.

Many studies [13–17] have already described poor prognostic factors. These include young age of onset of nephritis, African American ethnicity, hypertension, renal impairment at onset of LN, and poor pathologic findings on kidney biopsy [13–17]. A few studies have analysed whether the initial response to therapy predicts long-term renal outcome. Levey et al., found that treatment response was prognostic in a cohort of 63 patients with severe lupus nephritis [16]. Houssiau and colleagues have demonstrated that an early response to therapy at 6 months is the best predictor of good long-term renal outcome [13]. Our study also confirms that the long term renal outcome can be predicted by good response to therapy at 6 months.

From these results, we can confirm that the Euro-Lupus regimen is a suitable initial regimen for LN patients in SL. Use of alternative therapy in remission induction and maintenance is beneficial in patients who responded poorly to the Euro-lupus regimen.

There are some limitations in our study. This is a retrospective study in one race with a small sample size. A very selective and small number of patients were given MMF. Six patients who achieved partial remission were lost to follow up. In addition, steroid dosing was not uniform in all patients. These factors could have influenced the final result.

## Conclusions

The demographic and clinical features of WHO class III and IV LN in Sri Lankan patients were similar to that reported in the global literature. 75% of patients responded to the Euro-lupus regimen. Therefore, this regime is a suitable initial treatment for LN patients in SL. This study confirms that good long term renal outcome can be predicted by early response to therapy. Further studies are necessary to explore better treatment options for patients who fail to achieve remission during initial therapy.

## Abbreviations

CR: complete remission
ELNT: Euro lupus nephritis trial
LN: lupus nephritis
MMF: mycophenolate mofetil
PR: partial remission
SD: standard deviations
SL: Sri Lanka
SLE: systemic lupus erythematosus
